# Circulating tumour cell enumeration, biomarker analyses, and kinetics in patients with colorectal cancer and other GI malignancies

**DOI:** 10.3389/fonc.2023.1305181

**Published:** 2023-11-17

**Authors:** Walla Malkawi, Areeb Lutfi, Maaz Khan Afghan, Lamisha Mashiyat Shah, Lillian Costandy, Arturo B. Ramirez, Thaddeus C. George, Fatima Toor, Aliasger K. Salem, Pashtoon Murtaza Kasi

**Affiliations:** ^1^ Division of Pharmaceutics and Translational Therapeutics, University of Iowa, Iowa, IA, United States; ^2^ Division of Hematology and Oncology, Weill Cornell Medicine, New York, NY, United States; ^3^ RareCyte, Inc., Seattle, WA, United States; ^4^ Experimental Therapeutics Program, Holden Comprehensive Cancer Center, University of Iowa Hospitals and Clinics, Iowa, IA, United States; ^5^ Department of Electrical and Computer Engineering, University of Iowa, Iowa, IA, United States

**Keywords:** circulating tumor (ctDNA), circulating tumor cell (CTC), feasibility, gastrointestinal malignancies, biomarker, kinetics

## Abstract

**Objective:**

Most of the work in terms of liquid biopsies in patients with solid tumors is focused on circulating tumor DNA (ctDNA). Our aim was to evaluate the feasibility of using circulating tumor cells (CTCs) in peripheral blood samples from patients with advanced or metastatic gastrointestinal (GI) cancers.

**Methods:**

In this prospective study, blood samples were collected from each patient in 2 AccuCyte^®^ blood collection tubes and each tube underwent CTC analysis performed utilizing the RareCyte^®^ platform. The results from both tubes were averaged and a total of 150 draws were done, with 281 unique reported results. The cadence of sampling was based on convenience sampling and piggybacked onto days of actual clinical follow-ups and treatment visits. The CTC results were correlated with patient- and tumor-related variables.

**Results:**

Data from a total of 59 unique patients were included in this study. Patients had a median age of 58 years, with males representing 69% of the study population. More than 57% had received treatment prior to taking blood samples. The type of GI malignancy varied, with more than half the patients having colorectal cancer (CRC, 54%) followed by esophageal/gastric cancer (17%). The least common cancer was cholangiocarcinoma (9%). The greatest number of CTCs were found in patients with colorectal cancer (Mean: 15.8 per 7.5 ml; Median: 7.5 per 7.5 ml). In comparison, patients with pancreatic cancer (PC) had considerably fewer CTCs (Mean: 4.2 per 7.5 ml; Median: 3 per 7.5 ml). Additionally, we found that patients receiving treatment had significantly fewer CTCs than patients who were not receiving treatment (Median 2.7 versus 0.7). CTC numbers showed noteworthy disparities between patients with responding/stable disease in comparison to those with untreated/progressive disease (Median of 2.7 versus 0). When CTCs were present, biomarker analyses of the four markers human epidermal growth factor receptor 2 (HER2)/programmed death-ligand 1 (PD-L1)/Kiel 67 (Ki-67)/epidermal growth factor receptor (EGFR) was feasible. Single cell sequencing confirmed the tumor of origin.

**Conclusion:**

Our study is one of the first prospective real-time studies evaluating CTCs in patients with GI malignancies. While ctDNA-based analyses are more common in clinical trials and practice, CTC analysis provides complementary information from a liquid biopsy perspective that is of value and worthy of continued research.

## Introduction

The global cancer burden has been on the rise and amounts to an annual mortality count of 10 million (1). GI malignancies are one of the most prevalent cancer types accounting for 26% of the global incidence and 35% of total cancer deaths (2). The high mortality rates seen in GI cancers are caused by their ability to spread to far-off organs including the liver and lungs (3). By creating innovative techniques that aid in the early detection of cancer and the monitoring of disease development, there is a significant opportunity to increase survival rates.

Currently, the gold standard for diagnosis of cancer is a tissue biopsy. These tissue samples then undergo a histopathological evaluation for confirmation and tumor grading. For staging purposes, CT scans and MRIs are used to get radiological images to understand the size and relation of cancer to surrounding structures. Finally, PET scans are used to identify sites of distant metastases (4, 5). Until now clinical decisions regarding therapies have been based on these findings. However, there is a lag between cancer development and diagnosis. CT scans and MRIs often fail to pick up cancerous lesions unless they are a certain size and lack the ability to diagnose micrometastasis (6). A unique problem with cancer is the heterogenicity found in cancer cells which makes it challenging to judge cell types based on one biopsy sample (7). Biopsies can also not be relied on to monitor progression as repeat biopsies are invasive and unnecessary. Tumor markers, such as Cancer Embryogenic Antigen (CEA) have been used in colorectal cancer (CRC) for assessing disease progression and therapeutic response. However, it has a low sensitivity and specificity when it comes to diagnosing CRC (8). As evident, there is much to be done and studied to help clinicians diagnose, treat, and monitor CRC and other GI malignancies.

Liquid biopsy is a novel non-invasive method for evaluating cancer. It involves the screening of blood samples for circulating free DNA (cfDNA) and circulating tumor cells (CTCs) (9). In contrast to cfDNA analysis which analyzes a mixture of normal and tumor DNA strands free floating in circulation, CTC analysis looks at the entire and pure cancer cell and can provide additional information on the tumor pathology (10). CTCs are cancer cells that have shed off from the primary tumor or metastasis and have entered the circulation. Tumor cell dissemination is a vital process in cancer genesis and paves the way for metastasis (11).

A more accurate knowledge and classification of GI malignancies has resulted from the application of molecular biology to this field (12). It is crucial to understand the anomalies associated with oncogenes, tumor suppressor genes, cell adhesion molecules, and cell cycle regulators. Furthermore, changes in growth factors and cytokines as well as genetic instability add to the intricate processes underlying the development of GI malignancies (13). Keeping in line with cancer heterogeneity, CTC analysis has the unique potential to sample different cell types and be subsequently analyzed (14). Therefore, CTCs can help fill a void in non-invasive blood monitoring, as well as understanding the tumor biology and mechanisms of resistance to therapy (10). Enumerating CTCs has been proven to provide prognostic information, can show signs of early spread and hence act as a predictive marker for tumor recurrence (15). Additionally, CTCs can serve as real-time indicators of treatment efficacy and as a tool for monitoring tumor progression or response to treatment. CTCs are a promising noninvasive diagnostic and prognostic marker in metastatic and non-metastatic CRC in a number of studies (16–18). Most of the work pertaining to liquid biopsies is currently focused on ctDNA, and work on CTCs is less common. In this study of patients with GI malignancies, we set out to demonstrate the feasibility of CTC enumeration, biomarker analysis, and serial kinetics of CTCs in response to therapy.

## Materials and methods

**Figure 1:**
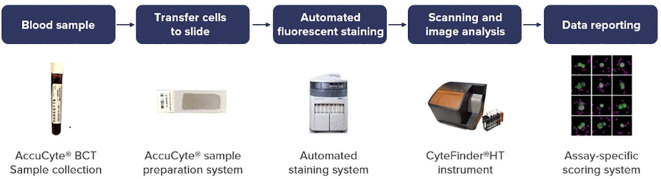
Scheme of comprehensive sample-to-results workflow of RareCyte^®^ platform.

In this prospective study, CTCs were detected and subjected to analysis in patients with metastatic gastrointestinal cancer, including colorectal cancer, pancreatic cancer, esophageal/gastric cancer, and cholangiocarcinoma as shown in **Figure 1**. CTCs were identified using the RareCyte^®^ platform, which relies on separation based on CTCs density (19). The Institutional Review Board (IRB) at the University of Iowa Holden Comprehensive Cancer Center reviewed this study and written informed consent was taken from all patients (n=59). Each patient’s blood sample was collected in two tubes, and each tube underwent CTC analysis. A total of 150 draws were performed, with 281 results recorded, and the average of the values from both tubes was calculated.

Analyses were performed within 96 hours of blood collection and results were discussed the same week. The workflow for liquid biopsy to data reporting is shown in **Figure 1**. Information regarding patient demographics and date of blood collection was available for all patients. CTCs were identified and enumerated based on standard definitions of nuclear/epithelial markers as previously reported (20, 21). When CTCs were present, biomarker analyses of the four markers HER2/PD-L1/Ki-67/EGFR were performed. In recent years these markers have been an area of focus for intense drug development in GI cancers and therefore the ability to assess them can prove to be clinically useful. Additionally, confirmation with panel-based cell sequencing was done. The aim of this study was to enumerate CTCs in peripheral blood samples of patients with GI malignancies. Additionally, we also aimed to correlate the CTC enumeration with the cancer type, treatment status, and tumor progression.

### Statistical analysis

The statistical tests and graphs were created using GraphPad PRISM (GraphPad, San Diego, CA). For the comparison of two groups and more than two groups, the Mann-Whitney test and Kruskal-Wallis test were used, respectively. Mean, standard deviation and range were used to represent the distribution of each continuous variable. Frequencies and percentages were used to represent categorical variables. Values are presented as median with interquartile range (IQR), and statistical significance was defined as a p-value <0.05.

## Results

This study included 59 patients, with 150 blood samples collected and 281 reported. The median age was 58 years, with 69% of the study population comprising of males. More than 57% had received treatment prior to taking blood samples (**Table 1**).

**Table 1:** Patient characteristics including age, gender and treatment received prior to taking blood samples.

Baseline features		
Age (years)	Median	58
	Mean	57.3± 12.2
Gender (n)	Female	18
	Male	41
Treatment received (n)	No	25
	Yes	34

**Figure 2:**
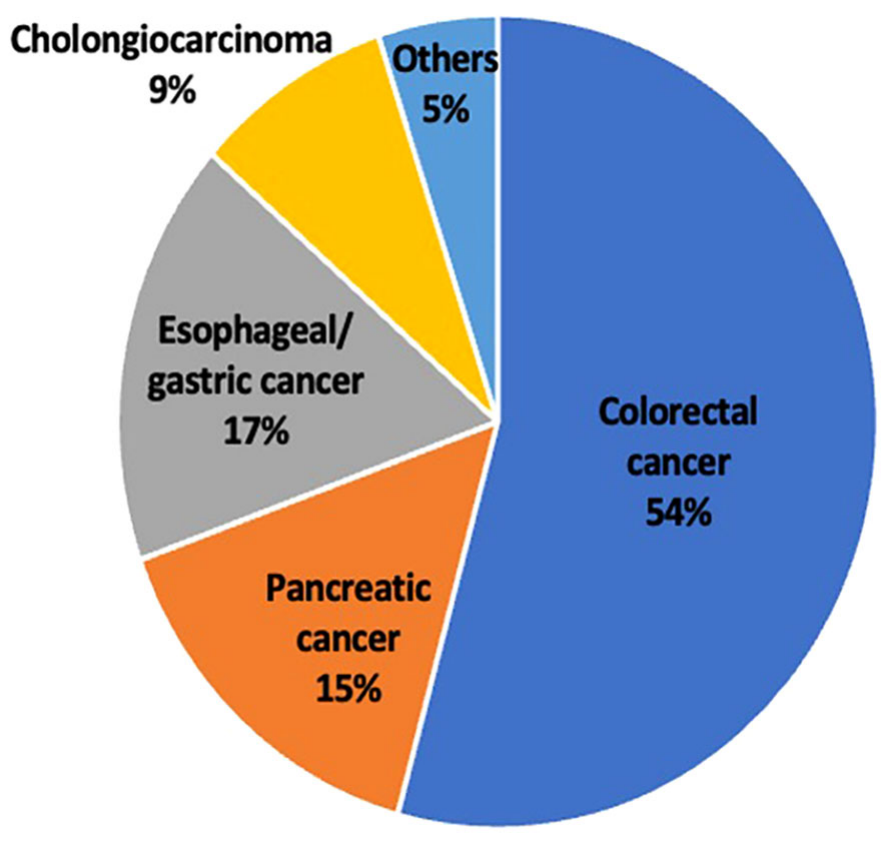
Pie chart showing the percentage of patients in this study with each tumor type.

The type of GI malignancy varied with more than half the patients having colorectal cancer (54%) followed by esophageal/gastric cancer (17%). The least common cancer was cholangiocarcinoma (9%) along with other types of GI malignancies which were grouped together (**Figure 2**).

**Figure 3:**
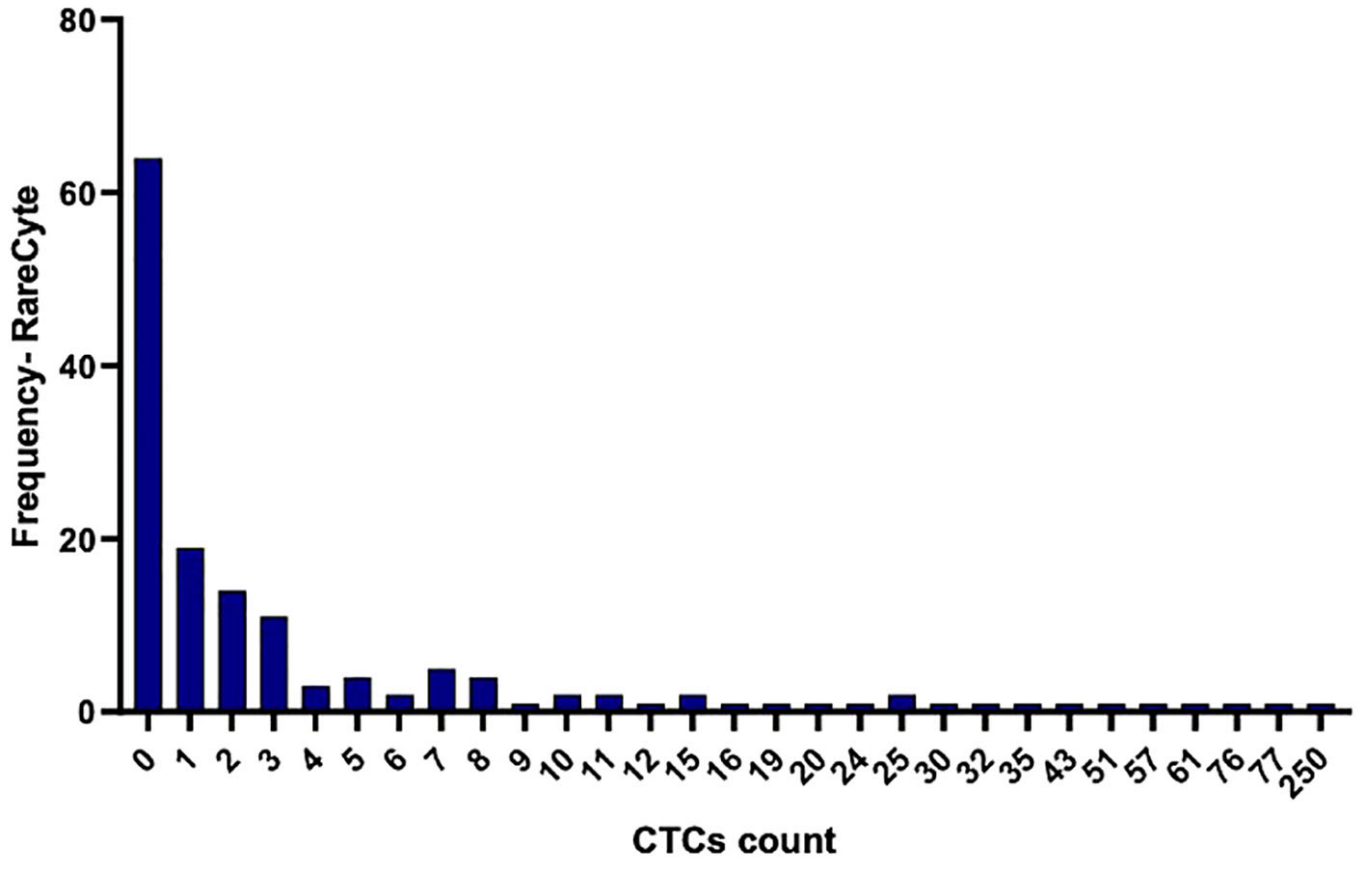
Histogram illustrating the frequency of distribution of detected CTCs per 7.5ml in the collected blood samples using the RareCyte platform.

**Figure 4:**
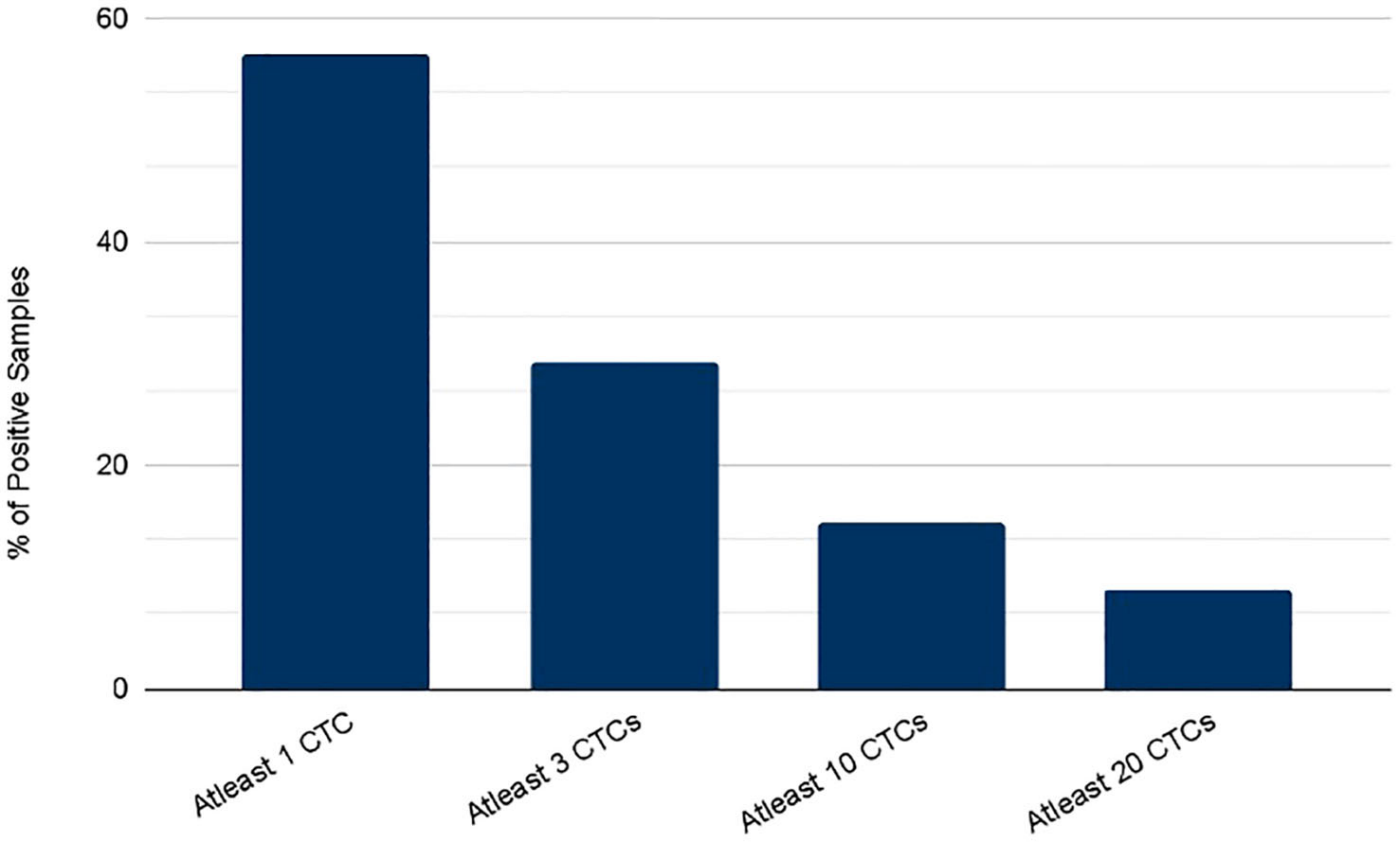
Percent of patients with a CTC count higher than each of the indicated cutoff values as determined using either the RareCyte system.

CTCs were detected in blood samples from patients with colorectal, pancreatic, esophageal/gastric cancer, or cholangiocarcinoma cancer patients. CTCs cells were nucleus+, epithelial markers+, and CD45-. Detecting CTCs using the RareCyte^®^ platform (**Figure 3**) indicated that the majority of patient’s samples (70.9%) contained < 3 CTCs while a small percentage of patient samples (4%) had > 50 CTCs. The percent of patients having a CTC count higher than each cut-off value (cut-off values were ≥ 1 cell, ≥ 3 cells, ≥ 10 cells, and ≥ 20 cells) is shown (**Figure 4**). ≥ 1 CTC was detected in 84 of 148 (56.8%), ≥ 3 CTCs were detected in 43 of 148 (29.1%) samples, ≥ 10 CTCs were detected in 22 of 148 (14.9%) samples, and ≥ 20 CTCs were detected in 13 of 148 (8.8%) samples.

**Figure 5:**
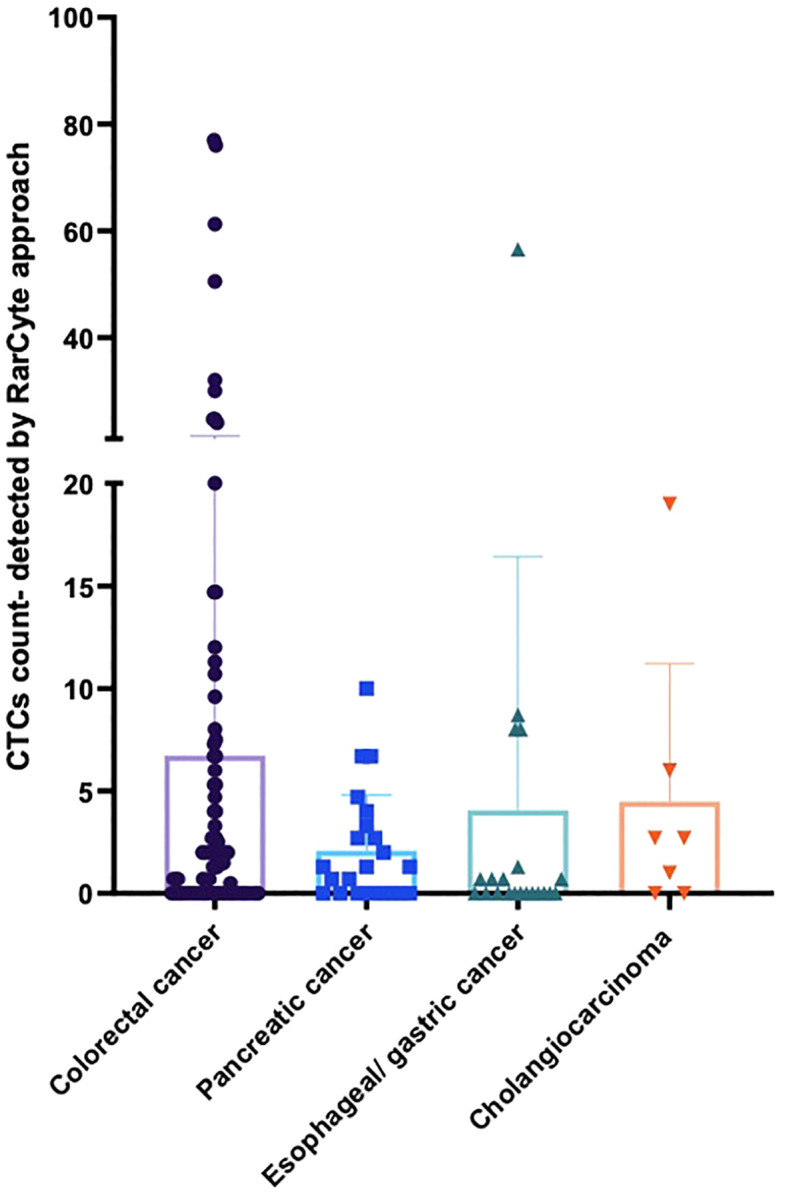
Average number of CTCs detected for patients based on tumor type per 7.5ml of patient blood sample. For patients with colorectal cancer (n = 90), pancreatic cancer (n = 23), esophageal/gastric cancer (n = 21) and cholangiocarcinoma (n = 7).

The detection of CTC numbers in samples from patients with different types of cancer was recorded; the median numbers of CTCs detected were [1.4 (IQR: 0 - 5.475; mean 6.73 (± 14.79)], [1.3 (IQR: 0- 3.3); mean 2.091 (± 2.72)], [0 (IQR: 0- 1); mean 4.06 (± 12.36)], and [2.7 IQR: 0- 6); mean 4.49 (± 6.73)] for colorectal cancer, pancreatic cancer, esophageal/gastric cancer, and cholangiocarcinoma, respectively (**Figure 5**).

**Figure 6:**
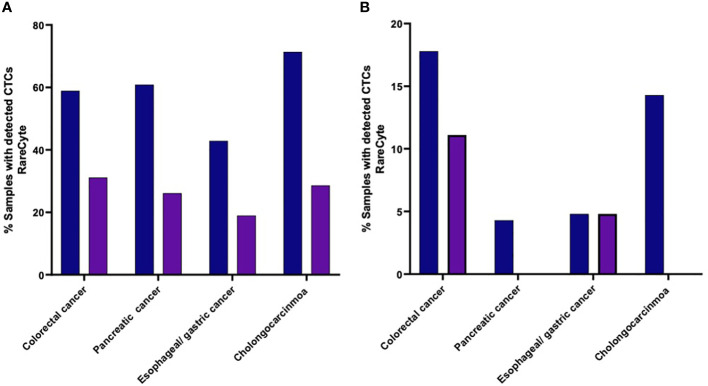
**(A, B)**. Percent of patients of each cancer type with a CTC count higher than the indicated cutoff values. **(A)** (≥ 1 CTC or ≥ 3 CTCs); **(B)** (≥ 10 CTCs or ≥ 20 CTCs).

The percent of patients of each cancer type that had a CTC count higher than each of the designated cutoff values (i.e. ≥ 1 cell, ≥ 3 cells, ≥ 10 cells, and ≥ 20 cells) were found to be different with different tumor types (**Figure 6**). Samples from colorectal cancer showed detectable CTCs at all the cutoff values which was not the case for other tumor types. At least 1 CTC (**Figure 6A**) was detected in 53 of 90 (58.9%), 14 of 23 (60.9%), 9 of 21 (42.9%), and 5 of 7 (71.4%) in colorectal cancer, pancreatic, esophageal/gastric, and cholangiocarcinoma cancer samples, respectively. At least 3 CTCs were detected in 28 of 90 (31.1%), 4 of 21 (19.0%), 6 of 23 (26.1%), and 2 of 7 (28.6%) in colorectal cancer, pancreatic, esophageal/gastric, and cholangiocarcinoma cancer samples, respectively. Using the RareCyte^®^ platform (**Figure 6B**), at least 10 CTCs were detected in 16 of 90 (17.8%), 1 of 23 (4.3%), 1 of 21 (4.8%), and 1 of 7 (14.3%) in colorectal cancer, pancreatic, esophageal/gastric, and cholangiocarcinoma cancer samples, respectively. At least 20 CTCs were detected in 10 of 90 (11.1%), 0 of 23 (0%), 1 of 21 (4.8%), and 0 of 7 (0%) in colorectal cancer, pancreatic, esophageal/gastric, and cholangiocarcinoma cancer samples, respectively.

**Figure 7:**
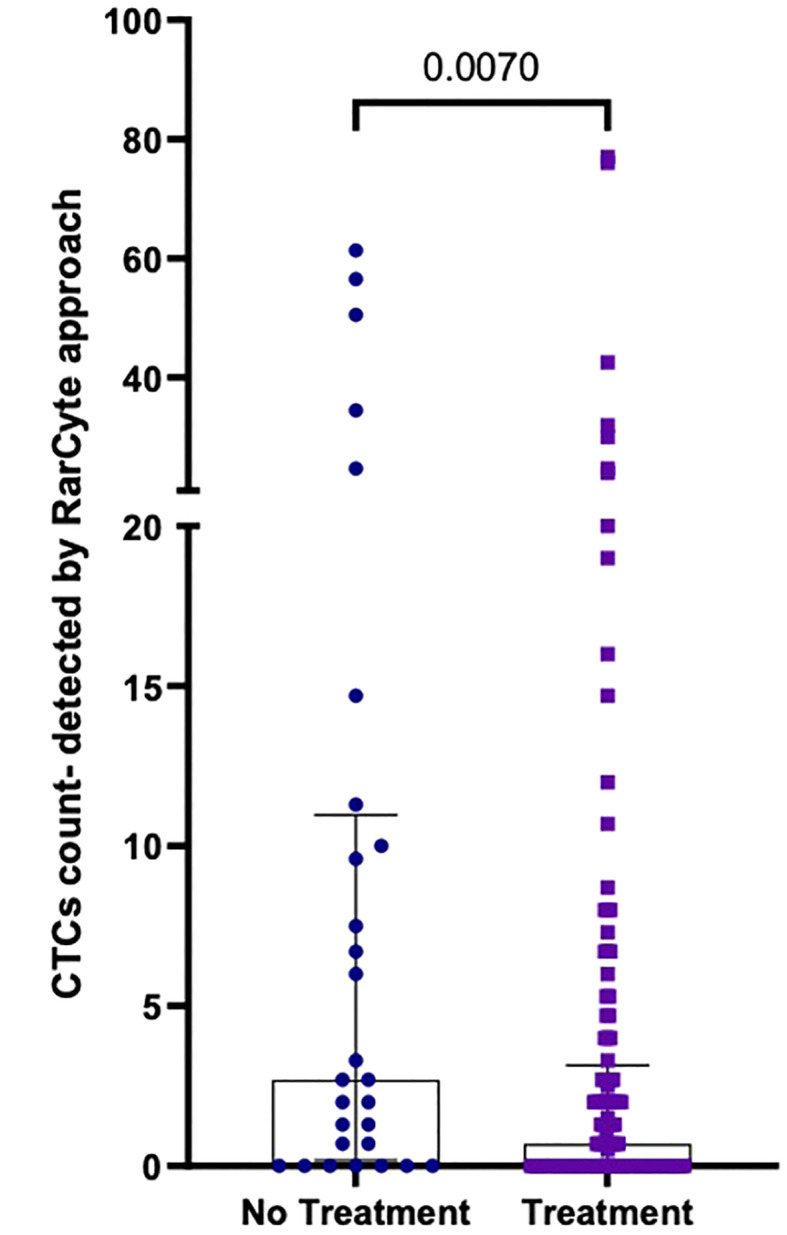
Number of CTCs per 7.5ml of patient blood samples from treated (n=120) versus untreated (n=28) patients; Values expressed in median (IQR), a significant difference was assessed with Mann-Whitney test.

The median number of CTCs detected (**Figure 7**) was determined for untreated patients [2.7 (IQR: 0.175- 10.98); mean 11.07 ( ± 17.83)] and treated patients [0.7 (IQR: 0- 3.15; mean 4.61 ( ± 11.71)]; a difference that was found to be statistically significant (p-value = 0.0070).

**Figure 8:**
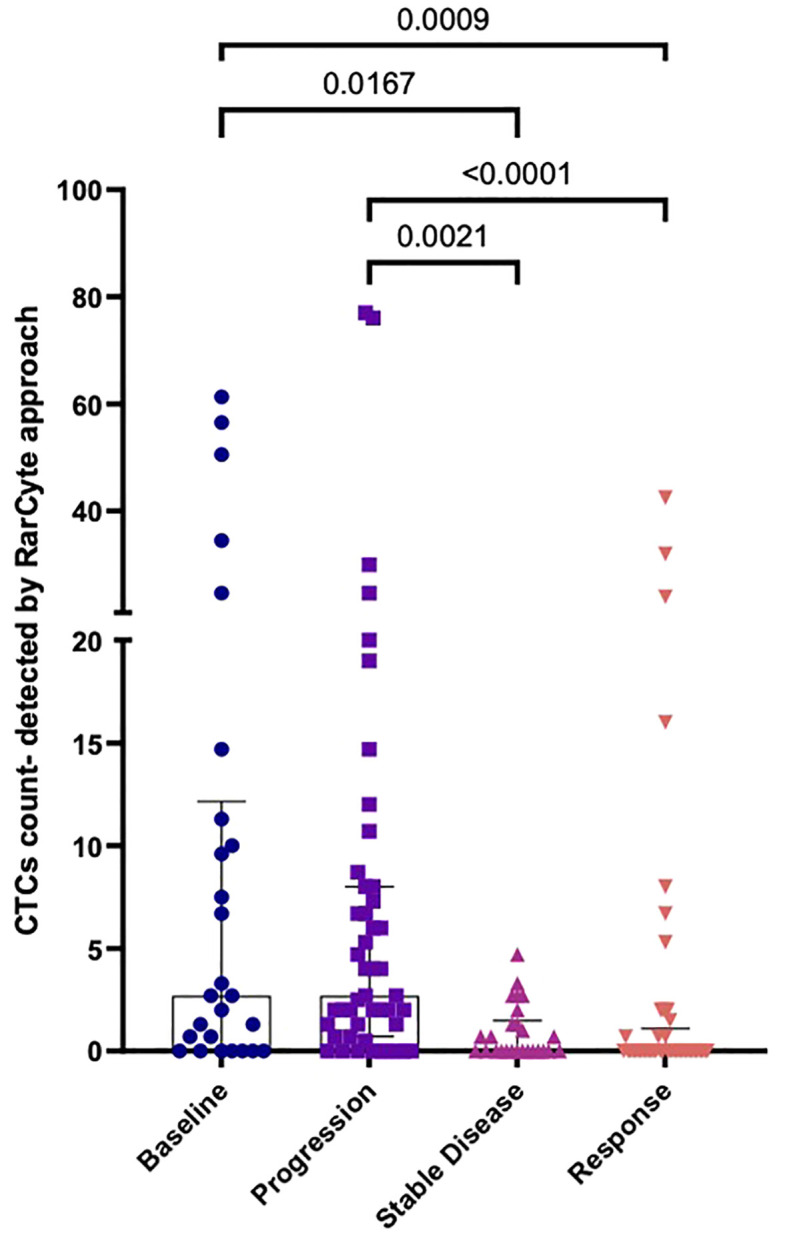
Numbers of CTCs per 7.5ml of patient blood samples based on treatment response. a) Median CTC numbers detected by RareCyte^®^ system for baseline (n= 26), progression group (n= 47), stable disease group (n= 26), and response group (n= 49). Values expressed as Median (IQR), a significant difference was assessed using the Kruskal-Wallis test.

Patient response to treatment was categorized into 4 groups: 1) Patients who were not on treatment before collecting blood samples (baseline group); 2) patients who showed no response to treatment (progression group); 3) patients who showed a response to treatment (response group); and 4) patients who demonstrated neither tumor progression nor regression during the course of treatment were (stable disease group). The median number of CTCs detected (**Figure 8**) was determined for the baseline group [2.7 (IQR: 0- 12.15); mean 11.62 ( ± 18.40)], the progression group [2.7 (IQR: 0- 8); mean 8.323 ( ± 15.99)], the stable disease group [0 (IQR: 0- 1.78); mean 0.9154 ( ± 1.31)], and the response group [0 (IQR: 0- 1.1); mean 2.982 ( ± 8.396)]. The difference in the number of CTCs in the baseline group was significantly higher than in the response group (p-value = 0.0009) and the stable disease group (p-value = 0.0167). Moreover, the difference in the number of CTCs in the progression group was significantly higher than in the response group (p-value < 0.0001) and the stable disease group (p-value = 0.0021).

**Figure 9:**
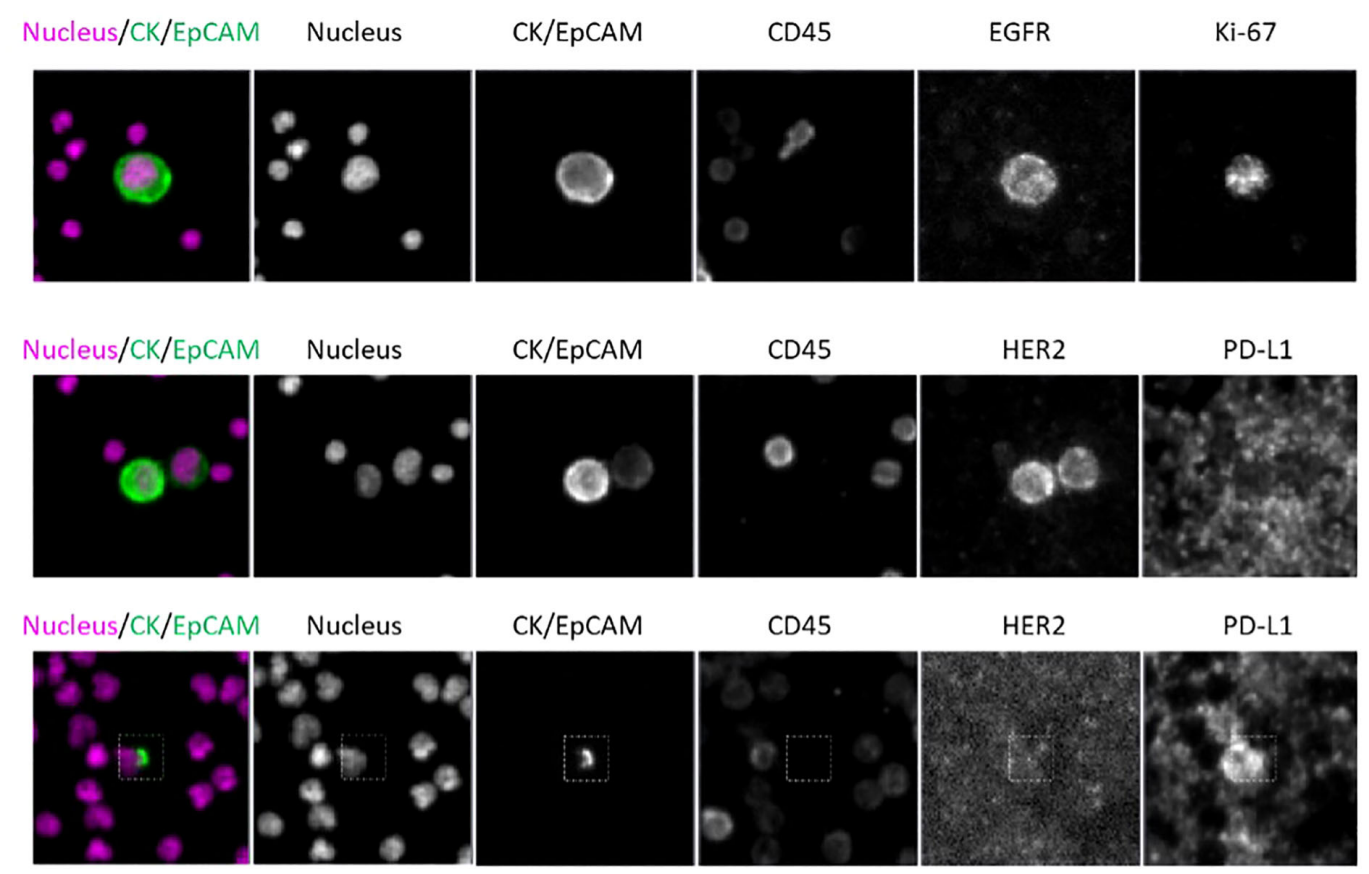
CTC assay for identification assay and biomarkers; Scale bar = 10 µm. Top: patient WT-022, Middle: patient WT-022, Bottom: patient RD-12.

Dual-biomarker CTC tests were utilized in the study to characterize firstly HER2 and PD-L1 and secondly, EGFR and Ki-67. Nuclear, epithelial (cytokeratin/EpCAM), and leukocyte (CD45) markers were used in each assay to identify CTCs, which were then evaluated for biomarker expression. Representative images (**Figure 9**) of CTCs from progressive cancer patients with biomarker testing are shown. The CTC in the upper panel exhibit EGFR expression on the cell membrane and nuclear Ki-67 expression, both signs of the proliferative phase. HER2 is expressed in both CTCs in a cluster shown in the middle panel. PD-L1 is expressed in the CTC in the lower panel. Note that PD-L1 is expressed by background platelets as well.

**Figure 10:**
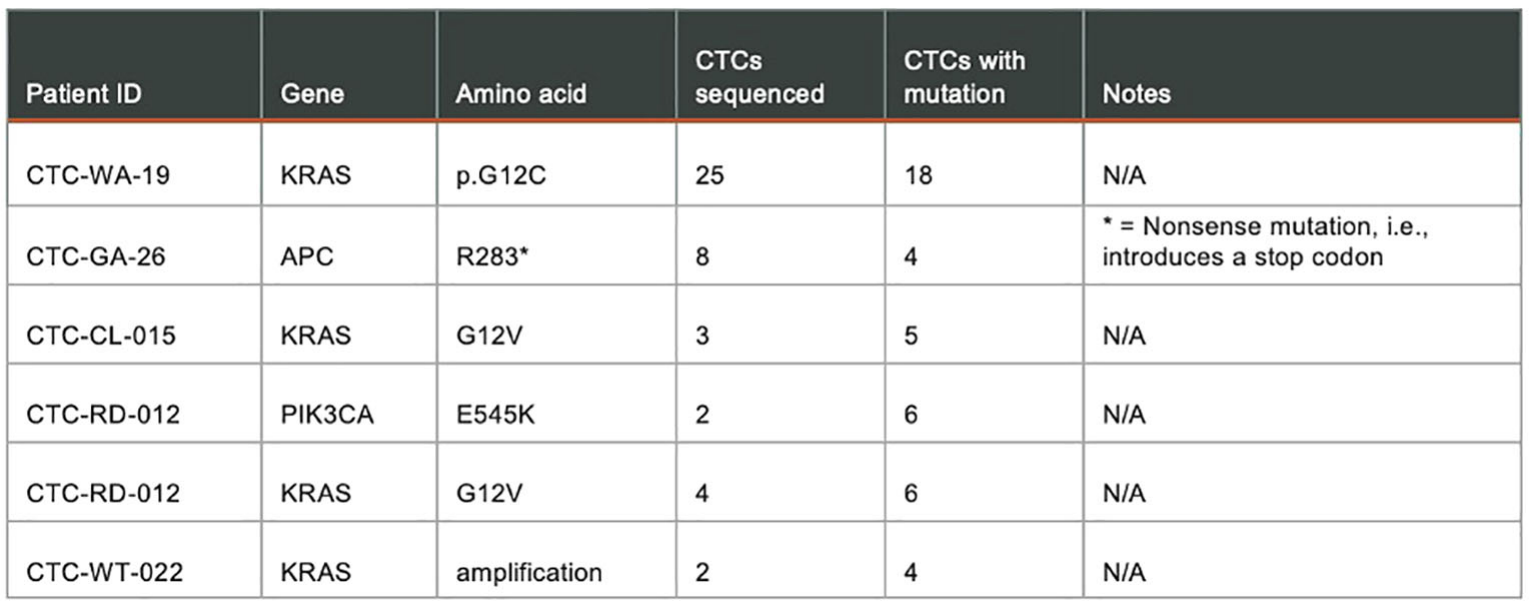
Targeted panel sequencing of individual CTCs from five cancer patients showing confirmed mutations detected.

**Figure 10**. shows results from a targeted panel sequencing of CTCs using a 65-gene cancer hotspot panel (CleanPlex OncoZoom Cancer Hotspot Kit, Paragon Genomics, Freemont CA) in five cancer patients. Using the RareCyte^®^ platform, blood was processed to slides, stained using a CTC enumeration assay or one of the dual biomarker assays shown in (**Figure 9**), and then scanned. After visualizing, the individual CTCs were isolated with the RareCyte CytePicker^®^ and deposited into PCR tubes for downstream targeted Next Generation Sequencing. For a particular mutation to be called, the following criteria must be met: 1) Non-synonymous substitution or indel; 2) catalogued in the COSMIC database; 3) present at ≥10% VAF (variant allele frequency) in at least two cancer sample libraries; 4) absent in germline control samples (WBCs); 5) coverage at variant locus is ≥100 reads for mutant samples and in at least one germline control; 6) not a common SNP, i.e. present in <1% of population based on gnomAD (genome aggregation database).

## Discussion

While most of the focus of current clinical trials and testing has been on ctDNA based assays, our study is a discovery platform showing the feasibility and value of CTC assessment in patients with GI malignancies. Furthermore, we were able to show pre-analytical variables e.g. patient- and tumor-related variables that can significantly impact the detection of CTCs.

There are various enrichment techniques to isolate CTCs. There are two major categories into which they can be divided: 1) Antigen-dependent (this uses the difference in expression of cell markers that are present in tumor cells but absent in other blood cells), 2: Antigen-independent (this uses the difference in biophysical properties between blood cells and CTCs while being agnostic to cellular markers) (22). The most commonly used and only FDA-approved technique, CellSearch is an antigen-dependent method and uses EPCAM to isolate cells and labeled Cytokeratin antibodies to identify them. However, the disadvantage is that this assay only provides recovery of CTCs with expression of both markers. Technologies, such as that developed by RareCyte, use the physical properties such as the density of nucleated cells in order to separate them from plasma and red blood cells. These nucleated cells are then separated using automated immunofluorescence microscopy to identify CTCs (19, 20, 23). Multimarker identification using EpCAM or Cytokeratin is a key advantage of the RareCyte platform in identifying these epithelial cells. It is important as most CTCs lie on a spectrum of the epithelial-mesenchymal transition (EMT). Cells that have undergone EMT switch from epithelial to mesenchymal markers, simultaneously expressing both phenotypes (24, 25). These EMT cells are linked to a poor prognosis and are important in the development of metastasis (26). Therefore, cells in EMT may possibly lose EpCAM but can still be detected by RareCyte assays due to the retention of cytoplasmic cytokeratin. This helps in expanding CTC enrichment techniques and better yields are achieved.

**Figure 11:**
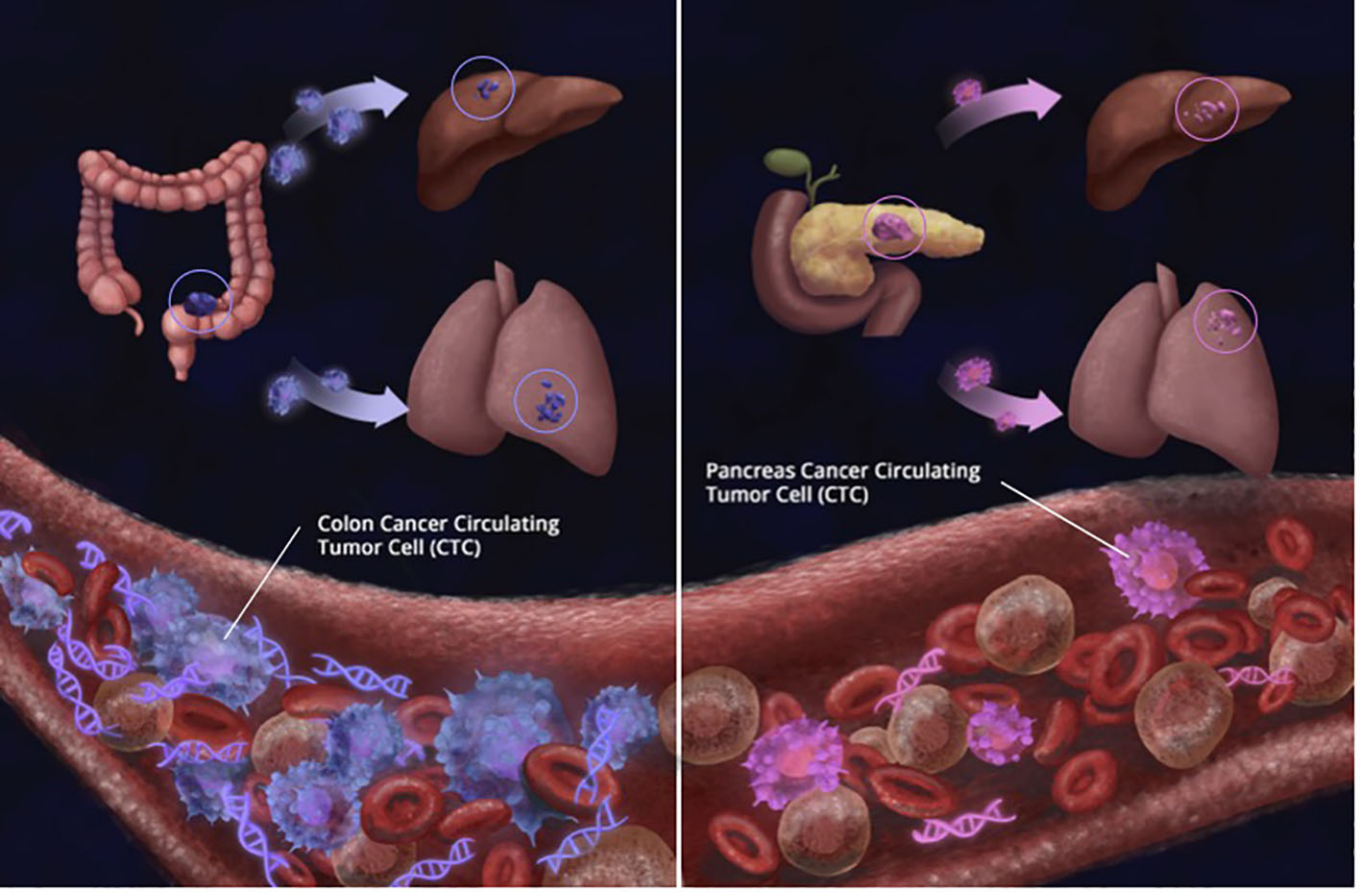
Simplistic illustration showing that regardless of tumor burden, patients with colorectal cancer had significantly more number of circulating tumor cells (CTCs) detected than patientswith pancreas cancer (CRC CTCs Mean: 15.8 per 7.5 ml; Median: 7.5 per 7.5 ml PC Mean: 4.2 per 7.5 ml; Median: 3 per 7.5 ml). Similarly, while at least 10 CTCs were detected in 16 of 90 (17.8%) CRC samples, only 1 of 23 (4.3%) patients with pancreas cancer had more than 10 CTCs detected; showing that biology and tissue type also matter for CTCs.

In this study, the greatest number of CTCs were found in patients with colorectal cancer. In comparison, patients with pancreatic cancer (PC) had considerably fewer CTCs, as seen in **Figure 11**. At least one-third of CRC patients had ≥ 3 CTCs. While no patients with PC had more than 20 CTCs, 17.4% and 11.4% of CRC patients had ≥ 10 and ≥ 20 CTCs, respectively. Our results are in concordance with other studies where it has traditionally been harder to isolate CTCs from pancreatic cancer. Ankeny et al. were able to report a median count of 2 CTCs in their PC cohort (27). In contrast, the median count of CTCs in colorectal cancer is higher across multiple studies. Drahomir et al. reported a median CTC count of 6.8 cells/ml in patients with CRC prior to surgery and 4.8 cells/ml post-surgery (28). The difference in results might be explained by the way CTCs are isolated, detected and/or defined. Most studies enumerating CTCs reported outcomes based on CellSearch and EpCAM-based CTC isolation. Despite the fact that 96% of pancreatic cancers are EpCAM-positive, EpCAM expression levels vary, with only half the tumors exhibiting strong expression (29, 30). This may help to explain the low number of patients with CTCs and also EpCAM-captured CTCs. However, in antigen-agnostic assays such as the RareCyte platform, the CTC isolation numbers are still low but have superiority in isolating CTCs as mentioned earlier (31).

Additionally, we found that patients receiving treatment had significantly fewer CTCs than patients who did not receive treatment (Median 2.7 versus 0.7).

There is limited literature available that explores the existence of CTCs in pancreatic cancer patients prior to and following neoadjuvant therapy. According to Poruk et al., there was no significant distinction between patients receiving or not receiving neoadjuvant therapy with regard to positive CTCs (32). In a study with 41 late-stage pancreatic cancer patients, it was found that before treatment with 5-fluorouracil (5-FU), 80.5% of the patient population had <2 CTCs, which had decreased to 29.3% following therapy administration (33). In a study pertaining to CRC, groups were stratified into those who received chemotherapy and those who did not. However, the CTCs were measured using two different techniques which showed conflicting results. In the group using the semi-automated cell imager, NYONE^®^, patients receiving chemotherapy had higher CTC counts occurring throughout the study duration and were significantly higher at one time point (Chemotherapy received: mean 7.75 cells, SD 14.17 *vs*. Chemotherapy not received: mean 0.75 cells, SD 0.5; p<0.05). However, within the same patient subset, when analyzed using CK-20 qt-PCR assay, patients who received chemotherapy showed lower relative CTC counts (34). The relevance of CTC detection prior to and following neoadjuvant therapy and its association with patient outcomes require additional research with larger patient populations.

Considering that CTCs can be more sensitive than imaging tests, researchers choose to use CTCs to assess the effectiveness of treatment interventions (35). We were able to demonstrate a relationship between tumor progression and CTC counts in patients with metastatic gastrointestinal cancer. CTC numbers showed noteworthy disparities between patients with responding/stable disease in comparison to those with untreated/progressive disease (Median of 0 versus 2.7). Multiple studies have been conducted in the past to elucidate the prognostic relevance of CTC counts in different GI malignancies. A meta-analysis conducted for metastatic CRC, including 1847 participants revealed that a high CTC number was a reliable and independent indicator of PFS prognosis (HR = 1.8; 95% CI 1.5-2.1) and OS (HR = 2; 95% CI 1.5-2.7) (18). Patients having pancreatic cancer who had less than 3 CTCs had significantly longer overall survival (OS) than those who had at least 3 or more CTCs (15.2 *vs*. 10.2 months, P<0.05). Elevated CTC count was a reliable predictor of worse OS, according to multivariate analysis (HR = 4.547, P<0.05) (36). Similarly, a meta-analysis with pancreatic cancer patients showed that there was a statistically significant hazard ratio difference in disease free survival and overall survival when CTCs were detected at diagnosis (HR = 1.93, 95% CI 1.19–3.11, *P*<0.05) and OS (HR = 1.84, 95% CI 1.37–2.45, *P* =< 0.05) (37). This highlights the potential use of CTCs as predictive markers among disease response groups.

While this is hypothesis generating, given the feasibility of assessing CTC enumeration and biomarker analyses that are possible due to the intact membrane structure of circulating tumor cells as opposed to ctDNA, CTCs may have more value for assessing cell-surface protein-based biomarkers such as HER2, PD-L1, and beyond. While anecdotal, it appears CTCs appear to clear the fastest from circulation as a measure of response to therapy (38). Similar to how liquid biopsies (ctDNA) tests are not meant to replace tissue-based genetic testing, CTCs here are not necessarily meant to replace ctDNA but be complimentary to it. There is also work done by other groups to show that since CTCs are viable, if enough of them are present, they can be grown to potentially develop cell lines and/or organoids for drug testing. Our work lays the foundation to formally integrate these novel biomarkers now that practical issues of same day analyses are no longer a barrier. For the current RareCyte platform, testing can be performed up to 14 days for a Clinical Laboratory Improvement Amendments (CLIA) CTC report, ideally within 7–8 days of collection. Turnaround time is also key and for CTC results it has been 3–5 days as opposed to around 7–9 days for most commercially available ctDNA platforms. It is also important to highlight that no special processing is required on the day of collection for CTC analysis for this particular platform and samples can be stored and shipped at room temperature. This opens up a plethora of possibilities and options where CTC evaluation may have more value as opposed to ctDNA-based collections. Lastly, at least for now, CTC evaluation is more suited for advanced/metastatic settings as opposed to some of the ctDNA/methylation-based platforms that are moving up the journey of a patient with cancer into the minimal residual disease and early detection setting. As to which type of liquid biopsy would be more of value (CTC versus ctDNA), it would depend on the setting, the cancer type, the timing of treatment, and the particular biomarker of interest.

## Limitations

Additional genomic, transcriptomic, and proteomic analysis may lead to a more in-depth characterization of CTCs, furthering their clinical use. This analysis can also help in identifying potential drug targets along with drug sensitivity testing. However, there are few ways to extract viable CTCs, and those that do also produce limited CTCs which makes culturing very difficult. This is in addition to the challenge of replicating optimal microcirculatory conditions to keep CTCs alive. Therefore, cancer cell lines derived from CTCs are lacking and will remain so unless culture conditions are improved. Finally, the analyses of CTCs should also involve phenotypic identification that is specifically suited to identify cells based on epithelial and mesenchymal markers because they are heterogeneous populations.

## Conclusion

Our study is one of the most extensive prospective studies on GI malignancies, demonstrating the viability and importance of analyzing CTCs. Our efforts of enumerating, serially monitoring, analyzing biomarkers, and sequencing CTCs will hopefully offer better insights to future investigators so that they may consider using CTCs in patients with GI malignancies. While ctDNA-based systems are currently the primary focus, CTCs provide a new perspective from a liquid biopsy aspect that is relevant yet worthy of future research. Future research will need to validate our findings using larger patient groups and extensive follow-up periods. In the context of enabling personalized therapy approaches for precision medicine, our efforts may assist in developing a longitudinal sample plan for liquid biopsies in the monitoring of cancer patients.

## Data availability statement

The original contributions presented in the study are included in the article/supplementary material. Further inquiries can be directed to the corresponding author.

## Ethics statement

The studies involving humans were approved by The Institutional Review Board (IRB) at the University of Iowa Holden Comprehensive Cancer Center. The studies were conducted in accordance with the local legislation and institutional requirements. The participants provided their written informed consent to participate in this study.

## Author contributions

WM: Conceptualization, Formal Analysis, Investigation, Methodology, Visualization, Writing – original draft, Writing – review & editing. AL: Visualization, Writing – original draft, Writing – review & editing. MA: Writing – review & editing. LS: Writing – review & editing. LC: Formal Analysis, Methodology, Visualization, Writing – review & editing. AR: Formal Analysis, Methodology, Visualization, Writing – review & editing. TG: Formal Analysis, Methodology, Visualization, Writing – review & editing. FT: Supervision, Writing – review & editing. AS: Methodology, Supervision, Writing – review & editing. PK: Methodology, Supervision, Writing – review & editing.
